# Andexanet alfa–induced heparin resistance during percutaneous coronary intervention for ST-elevation myocardial infarction: a case report

**DOI:** 10.1093/ehjcr/ytaf497

**Published:** 2025-10-03

**Authors:** Satoru Sasaki, Daisuke Matsumoto, Ryo Takeshige, Sachiyo Iwata

**Affiliations:** Division of Cardiovascular Medicine, Yodogawa Christian Hospital, 1-7-50 Kunijima, Higashiyodogawa-ku, Osaka, Osaka 533-0024, Japan; Division of Cardiovascular Medicine, Yodogawa Christian Hospital, 1-7-50 Kunijima, Higashiyodogawa-ku, Osaka, Osaka 533-0024, Japan; Division of Cardiovascular Medicine, Yodogawa Christian Hospital, 1-7-50 Kunijima, Higashiyodogawa-ku, Osaka, Osaka 533-0024, Japan; Division of Cardiovascular Medicine, Yodogawa Christian Hospital, 1-7-50 Kunijima, Higashiyodogawa-ku, Osaka, Osaka 533-0024, Japan

**Keywords:** Andexanet alfa, Heparin resistance, Percutaneous coronary intervention, STEMI, Anticoagulation management, Case report

## Abstract

**Background:**

Andexanet alfa is a recombinant modified factor Xa decoy protein used to reverse the anticoagulant effects of direct oral factor Xa inhibitors, including apixaban, rivaroxaban, and edoxaban. While effective in controlling life-threatening bleeding, it may induce heparin resistance, which complicates anticoagulation during procedures requiring urgent revascularization. Its potential to cause significant heparin resistance during percutaneous coronary intervention (PCI) has been reported only rarely and remains underrecognized. This phenomenon poses a major therapeutic dilemma when emergent PCI is required, especially in life-threatening settings.

**Case summary:**

An 82-year-old man on edoxaban for atrial tachycardia presented with a left iliac bone fracture and expanding haematoma. Andexanet alfa was administered to reverse anticoagulation. Shortly thereafter, he developed acute anterior ST-segment elevation myocardial infarction (STEMI). Emergency PCI revealed a new occlusion in the proximal left anterior descending artery. Despite receiving 30 000 units of unfractionated heparin, the activated clotting time reached only 249 s after a 36-min delay, consistent with heparin resistance. Intra-aortic balloon pumping and veno-arterial extracorporeal membrane oxygenation were required to stabilize haemodynamics. Although revascularization was successfully completed, the patient ultimately died from pneumonia on post-operative Day 21.

**Discussion:**

Although STEMI following andexanet alfa administration has been reported, this case underscores the additional challenge of profound heparin resistance compromising timely anticoagulation during emergent PCI. Rapid recognition of andexanet alfa–induced heparin resistance is crucial in urgent PCI settings to ensure effective anticoagulation and prevent procedural delays that may compromise outcomes.

Learning points

- Andexanet alfa–induced heparin resistance significantly delays activated clotting time during emergent percutaneous coronary intervention, even with escalated heparin dosages.

## Introduction

Andexanet alfa (Ondexxya, Portola Pharmaceuticals, San Francisco, CA) is a recombinant modified decoy protein derived from human factor Xa. It functions as a reversal agent for direct factor Xa inhibitors, including apixaban, rivaroxaban, and edoxaban, and is specifically indicated for patients experiencing life-threatening bleeding associated with these anticoagulants. Originally developed to counteract the effects of both direct and indirect factor Xa inhibitors, its current regulatory approval is limited to the reversal of direct factor Xa inhibitors.^1,2^

The ANNEXA-4 study provided evidence of its efficacy in reversing anticoagulant effects during major bleeding episodes.^2^ Andexanet alfa, however, has been reported to induce ‘heparin resistance’, a phenomenon documented in prior cases.^3,4^ This intolerance poses significant challenges during percutaneous coronary intervention (PCI) for ST-segment elevation myocardial infarction (STEMI), where maintaining the optimal activated clotting time (ACT) is critical. Consequently, PCI in patients who have received andexanet alfa presents a unique challenge due to the potential for heparin resistance, necessitating careful anticoagulation management.

In this report, we present one of the earliest documented cases of emergency PCI for STEMI following andexanet alfa administration, highlighting the anticoagulation challenges caused by heparin resistance and offering practical insights into its recognition and potential management strategies in emergent PCI.

## Summary figure

**Figure:**
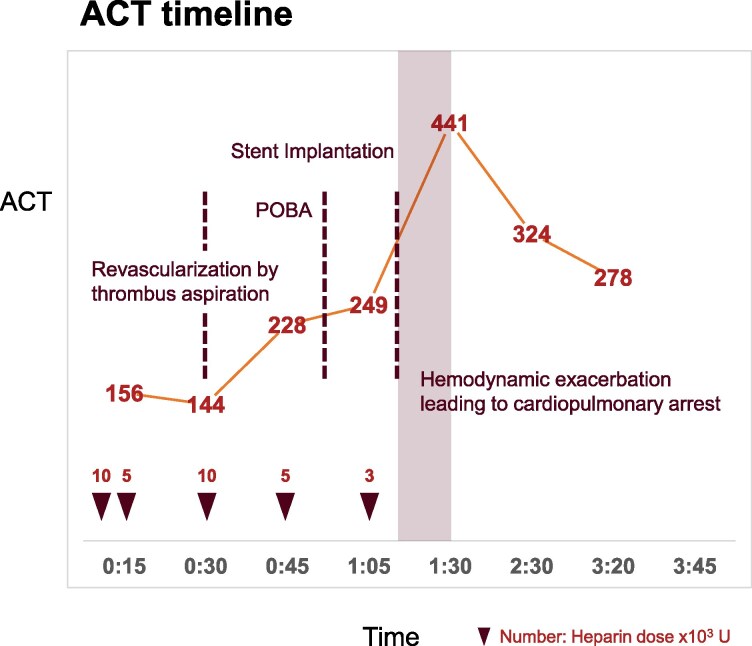
Time course of ACT following andexanet alfa administration and during PCI. The *y*-axis represents the ACT in seconds, while the *x*-axis denotes the elapsed time from the procedure. Key procedural events, including percutaneous coronary intervention steps such as plain old balloon angioplasty, stent implantation, and haemodynamic deterioration leading to cardiopulmonary arrest, are indicated. Despite incremental heparin dosing, ACT prolongation was delayed, reflecting heparin resistance associated with prior andexanet alfa administration. *y*-axis: ACT in seconds. *x*-axis: time elapsed from the start of PCI. Despite escalating heparin doses, ACT prolongation remained suboptimal. The significant ACT delay highlights the heparin resistance induced by prior andexanet alfa administration, complicating procedural anticoagulation.

## Case presentation

### Clinical history

The patient was an 82-year-old man with atrial tachycardia on anticoagulant therapy. His medical history included an inferior wall myocardial infarction (26 years ago) with unsuccessful revascularization of the right coronary artery and a posterior wall infarction (17 years ago) treated with bare-metal stent placement in the proximal left circumflex artery and an additional stent in the diagonal branch, with subsequent aspirin therapy.

Two years prior to presentation, he underwent a right middle lobectomy for lung cancer, but metastatic lesions were found in the brain, lungs, and adrenal gland 6 months post-operatively. He was receiving outpatient systemic chemotherapy and gamma knife therapy for brain metastases. His medication included aspirin 100 mg, edoxaban 15 mg, carvedilol 10 mg, atorvastatin 5 mg daily, and several gastric mucosal protectants. In the weeks prior to admission, the patient experienced exertional chest discomfort. Based on this, further evaluation for possible coronary ischaemia had been under consideration, and hospital admission for diagnostic work-up was being planned. Although there was no history of haematologic disorders or thrombophilia, the patient’s metastatic cancer may have contributed to a hypercoagulable state. There was no known history of haematologic disorders or thrombophilia. A prothrombotic state associated with metastatic cancer was considered the most likely contributor.

### Presentation and initial management

**Figure 1:**
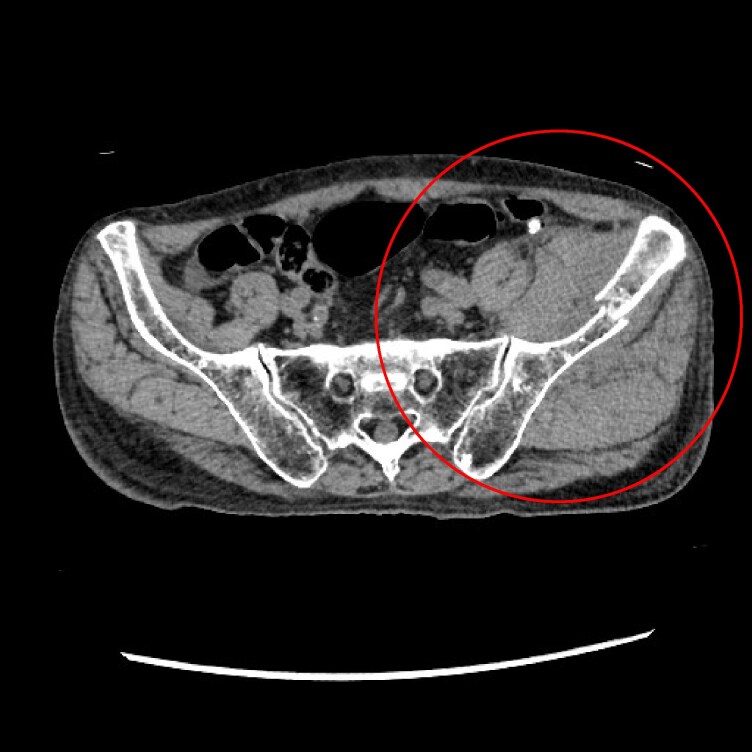
Contrast-enhanced computed tomography image showing a left iliac bone fracture with an adjacent expanding haematoma (highlited area).

The patient presented to the emergency department following sudden onset of buttock pain and difficulty in movement while changing clothes, with no history of trauma or fall. A computed tomography scan revealed a left iliac bone fracture with haematoma (*Figure 1*). Given his history of metastatic cancer, the fracture was presumed to be pathological rather than due to a mechanical injury. Blood tests revealed a haemoglobin level of 10.1 g/dL (normal range 131.–16.6 g/dL), a platelet count of 276 × 10^9^/L (normal range 130–349 × 10^9^/L), and a prothrombin time activity of 62% (normal range 80–100%). Additional laboratory findings are provided in the Supplementary material. Blood transfusion was not performed.

As only 6 h had passed since his last edoxaban dose, andexanet alfa was administered in the emergency room. Shortly after completion of the infusion, the patient developed sudden chest pain, and ST-segment elevation was observed on the electrocardiogram (see Supplementary material). Vital signs included a heart rate of 115 b.p.m., blood pressure of 86/65 mmHg, and SpO2 of 98% on 2 L/min oxygen. Physical examination revealed a systolic murmur, tenderness over the left buttock, a subcutaneous haematoma, and mild bilateral pedal oedema. Electrocardiography revealed ST-segment elevation in the anterior leads, suggesting an acute anterior wall myocardial infarction. Transthoracic echocardiography demonstrated severe global left ventricular hypokinesia with an estimated ejection fraction of <25% on visual assessment, along with severe mitral regurgitation (no record of images available).

### Coronary angiography and percutaneous coronary intervention

**Figure 2:**
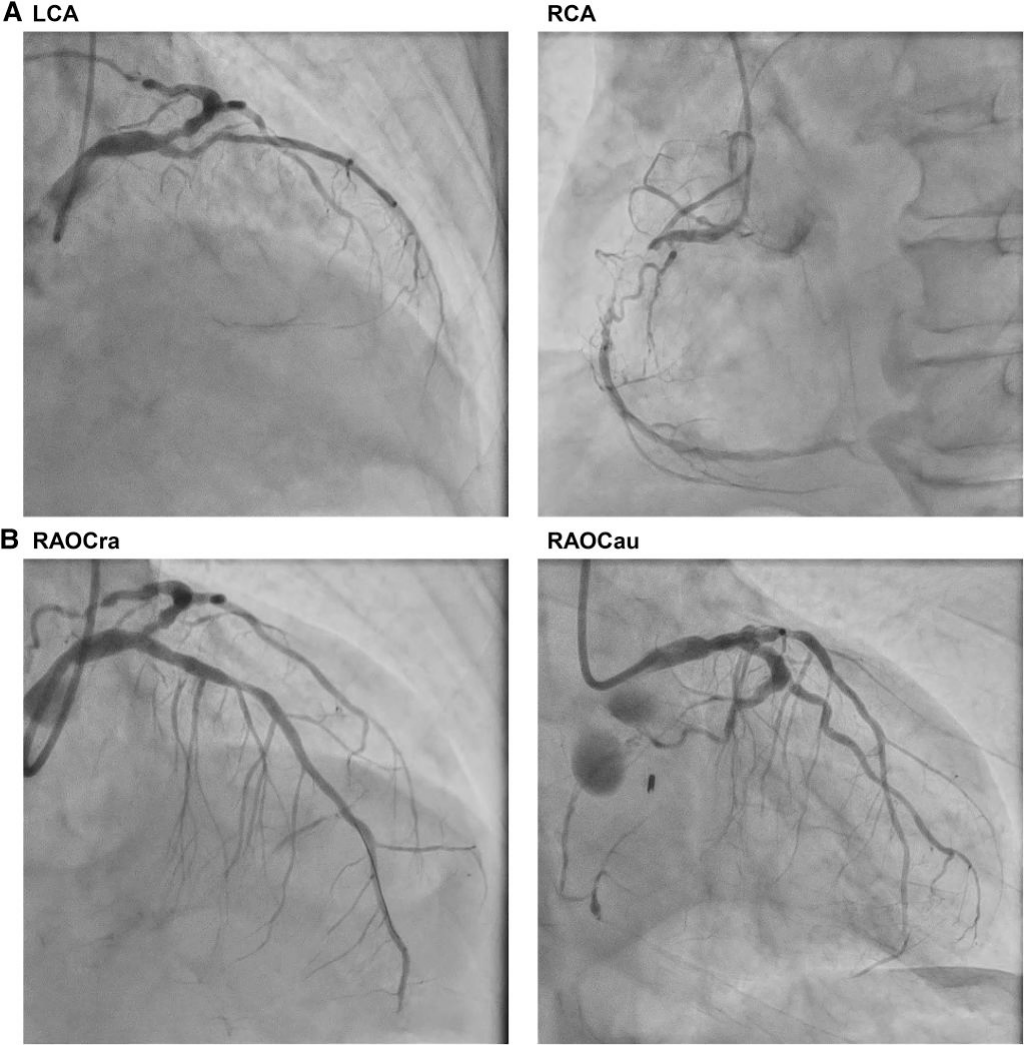
Coronary angiography before and after percutaneous coronary intervention. (*A*) Pre-procedural coronary angiography showing chronic total occlusion of the proximal right coronary artery and a new acute thrombotic occlusion in the proximal left anterior descending artery. (*B*) Post-procedural coronary angiography after successful percutaneous coronary intervention with thrombus aspiration and drug-eluting stent implantation in the left anterior descending artery, demonstrating restoration of Thrombolysis in Myocardial Infarction 3 flow.

In the catheterization laboratory, prasugrel (20 mg) was administered, in view of the need for rapid and potent platelet inhibition in the setting of anterior STEMI complicated by cardiogenic shock. Despite the patient’s elevated bleeding risk, the high thrombotic burden and haemodynamic instability were warranted aggressive antiplatelet therapy in this emergency context. Emergency coronary angiography was then performed following administration of the prasugrel loading dose and 10 000 units of unfractionated heparin (UFH). The angiographic findings revealed chronic total occlusion of the proximal right coronary artery and a new occlusion of the proximal left anterior descending (LAD) artery (*Figure 2A*).

Given the presence of cardiogenic shock, intra-aortic balloon pumping (IABP) was initiated via the right femoral artery before proceeding with PCI. Revascularization of the LAD was successfully achieved within 27 min using thrombus aspiration. Despite incremental heparin dosing, achieving an ACT of 249 s required 30 000 units of UFH, leading to a procedural delay of 36 min (Summary figure).

While thrombus aspiration and balloon dilatation temporarily achieved Thrombolysis in Myocardial Infarction (TIMI) 3 flow, re-occlusion occurred repeatedly. Therefore, a drug-eluting stent was implanted to secure sustained reperfusion (see Supplementary material).

During post-stenting dilation, the patient experienced recurrent episodes of haemodynamic instability, culminating in pulseless electrical activity. Veno-arterial extracorporeal membrane oxygenation was promptly initiated, and TIMI grade 3 flow was successfully restored (*Figure 2B*; Supplementary material).

Despite initial thrombus aspiration and ballooning, re-occlusion occurred repeatedly. Given the inadequate ACT prolongation and the critical importance of the anterior wall, these events likely precipitated the cardiopulmonary collapse.

### Post-operative course

The patient’s highest recorded levels of creatine kinase (CK) and CK-MB were 6011 IU/L and 451 IU/L, respectively. On post-operative day (POD) 2, transcatheter haemostasis was performed for an expanding pelvic haematoma. Veno-arterial extracorporeal membrane oxygenation was weaned on POD 5, followed by IABP removal on POD 8. Despite initial recovery, recurrent pneumonia led to the patient’s death on POD 21. Throughout the clinical course, there was no evidence suggestive of heparin-induced thrombocytopenia.

## Discussion

While STEMI after andexanet alfa administration has been previously reported,^5^ the procedural challenges—particularly those related to anticoagulation during PCI—remain underexplored. Our case highlights the clinical implications of heparin resistance in this urgent setting. Although this phenomenon has been increasingly recognized in the context of cardiopulmonary bypass (CPB) of cardiovascular surgery, no prior reports have described its impact in the setting of urgent PCI for STEMI.^3,4,6–11^

Andexanet alfa is a recombinant, modified human factor Xa decoy protein, approved for the reversal of direct oral factor Xa inhibitors—including apixaban, rivaroxaban, and edoxaban—in cases of life-threatening or uncontrolled bleeding.^1,2^ Its administration, however, has been associated with impaired response to UFH, due to its binding to the heparin-antithrombin (AT) complex, effectively neutralizing AT activity and thereby attenuating the anticoagulant effect of heparin.^12,13^

In cardiovascular surgery needing CPB, where ACT targets are >480 s, several reports have documented inadequate ACT prolongation despite massive UFH dosing.^3,4,9^ Rescue strategies such as AT supplementation, fresh frozen plasma, or use of direct thrombin inhibitors (e.g. argatroban) or serine protease inhibitors (e.g. nafamostat mesylate) have been reported with varying success.^6–8,10,11,14^ In contrast, PCI typically requires a lower ACT target (250–300 s), generally achieved with 70–100 U/kg of UFH.^15^ Nonetheless, in the present case, over 600 U/kg (30 000 units) of UFH was needed to reach an ACT of only 249 s, clearly indicating profound heparin resistance.

In previously published cases, AT administration has proven effective in restoring heparin sensitivity. For instance, Apostel *et al*.^6^ described a case where ACT increased to >999 s following administration of 1000 units of AT after failure of high heparin dosing. Nagashima *et al*.^14^ similarly reported partial ACT recovery after the combined use of AT, argatroban, and nafamostat mesylate, in the setting of type A aortic dissection surgery. These findings are consistent with the pathogenesis of andexanet alfa–induced heparin resistance.^12^

**Figure 3:**
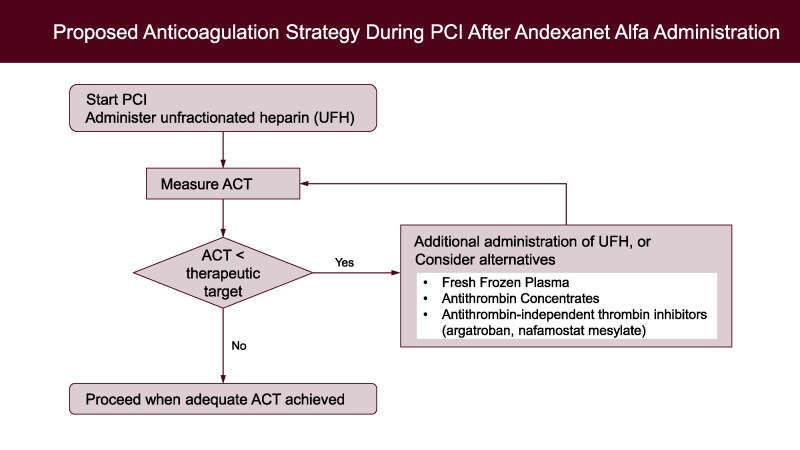
Proposed anticoagulation strategy during percutaneous coronary intervention after andexanet alfa administration, in cases where unfractionated heparin fails to achieve target activated clotting time due to heparin resistance, adjunctive strategies—such as administration of antithrombin, fresh frozen plasma, or direct thrombin inhibitors (e.g. argatroban or nafamostat mesylate)—may be considered. There are currently no clinical reports supporting the use of low-molecular-weight heparin, fondaparinux, or bivalirudin in this setting.

In our case, due to the emergent clinical situation, the limited manpower, and the lower ACT target for PCI compared with CPB, alternative anticoagulation strategies were not implemented. However, the 36-min procedural delay may have contributed to haemodynamic instability. Early recognition of the andexanet alfa–induced heparin resistance and prompt intervention with adjunctive strategies might have mitigated this clinical deterioration (*Figure 3*).

## Conclusion

This case highlights the challenges of managing andexanet alfa–induced heparin resistance during emergent PCI. Future research should aim to establish standardized anticoagulation strategies to mitigate heparin resistance following andexanet alfa administration. A deeper understanding of the interaction between andexanet alfa and anticoagulants is essential for developing effective protocols to prevent and manage this rare but life-threatening complication during emergent PCI.

## Lead author biography


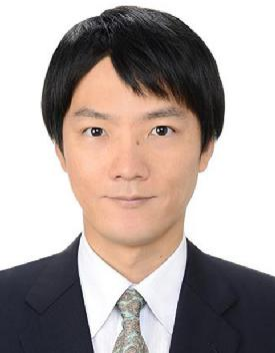


Dr Satoru Sasaki is a cardiologist and interventionalist at Yodogawa Christian Hospital, Japan.

## Supplementary Material

ytaf497_Supplementary_Data

## Data Availability

De-identified data are available upon request from the corresponding author.

## Supplementary material

**Consent:** Written informed consent for the publication of this case report, including accompanying images and clinical details, was obtained from the patient’s family. The authors confirm that this report complies with the ethical guidelines and policies of the journal.

**Funding:** The authors received no financial support for the research, authorship, or publication of this article.
