# Supplementary material for: Andexanet alfa–induced heparin resistance during percutaneous coronary intervention for ST-elevation myocardial infarction: a case report
Source: Eur Heart J Case Rep. 2025 Oct 3;9(10):ytaf497. doi: 10.1093/ehjcr/ytaf497 (PMC12548570; doi:10.1093/ehjcr/ytaf497)
Supplement: ytaf497_Supplementary_Data [file ytaf497_supplementary_data.zip › EHJCR_Supplementary_Materials.docx]

**Supplementary Materials**

**Supplementary Table: Laboratory Findings**

| **Test Item** | **Measured Value** | **Reference Range** | **Unit (SI)** |
| --- | --- | --- | --- |
| WBC | 120 | 32-85 | x10^9^/L |
| RBC | 3.96 | 400-539 | x10^12^/L |
| Haemoglobin | 10.1 | 13.1-16.6 | g/dL |
| Haematocrit | 32.8 | 38.5-48.9 | % |
| Platelet count | 276 | 13-34.9 | x10^9^/L |
| PT | 62 | 80-100 | % |
| aPTT | 37.7 | 24-39 | sec |
| Fibrinogen | 377 | 150-400 | mg/dL |
| D dimer | 6.19 | 0-0.99 | μg/mL |
| AST | 27 | 0-30 | U/L |
| ALT | 15 | 0-30 | U/L |
| gamma GTP | 27 | 0-50 | U/L |
| total bilirubin | 0.96 | 0.3-1.2 | mg/dL |
| ALP | 70 | 38-113 | U/L |
| LDH | 334 | 106-220 | U/L |
| total protein | 5.4 | 6.5-8.0 | g/dL |
| albumin | 3 | 4-5.2 | g/dL |
| BUN | 20.8 | 7-24 | mg/dL |
| Cr | 1.79 | 0-1 | mg/dL |
| Na | 141 | 136-147 | mmol |
| K | 2.5 | 3.6-5 | mmol |
| Cl | 100 | 98-109 | mmol |
| blood glucose | 6.2 | 3.9-5.6 | mmol |
| CRP | 3.11 | 0-0.29 | mg/dL |
| BNP | 1240 | 0-18.4 | pg/mL |

**Supplementary Figures**

**Supplementary Figure 1. Chest X-ray image in emergency room**


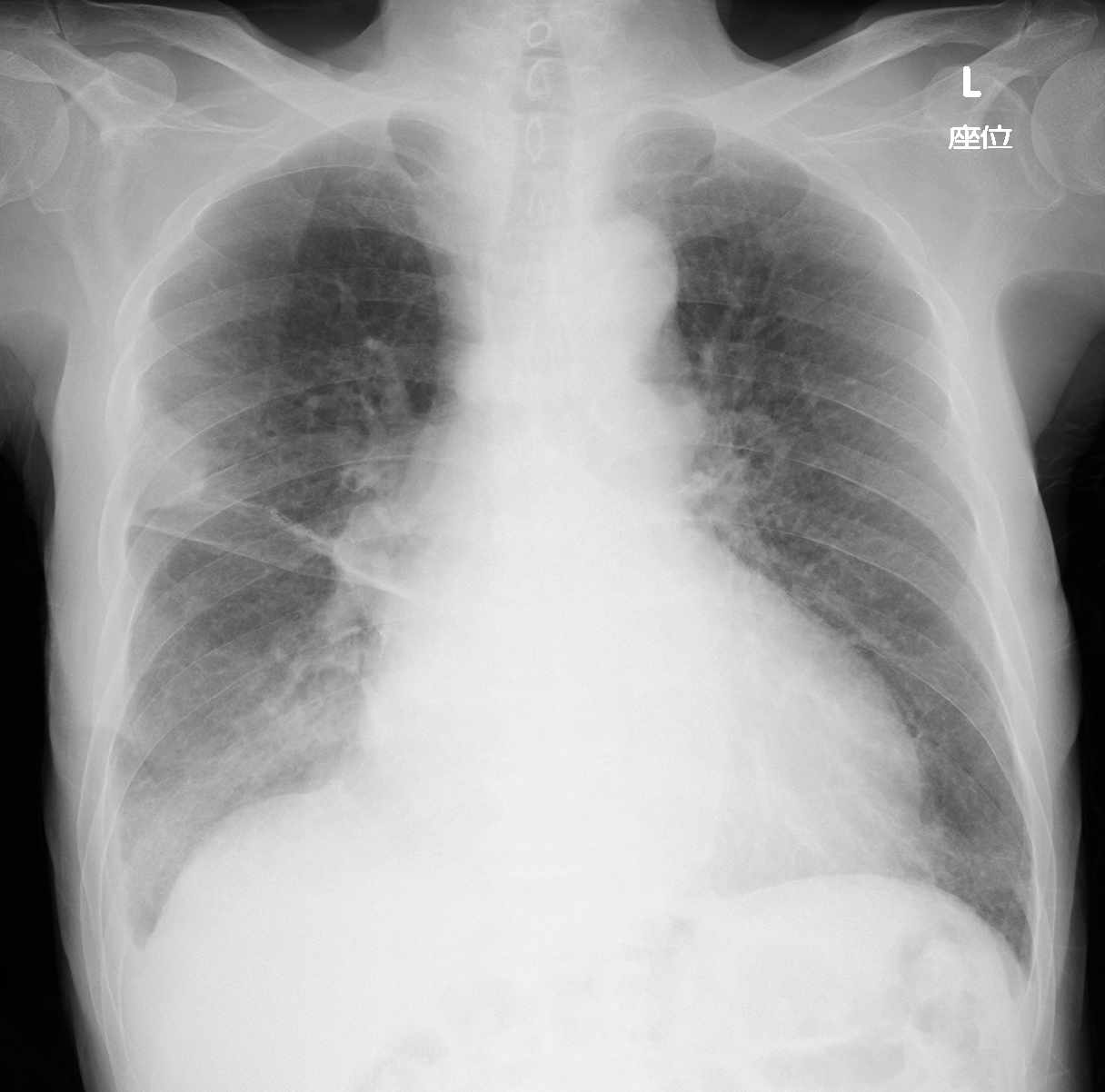


**Supplementary Figure 2. ECG in emergency room**

**
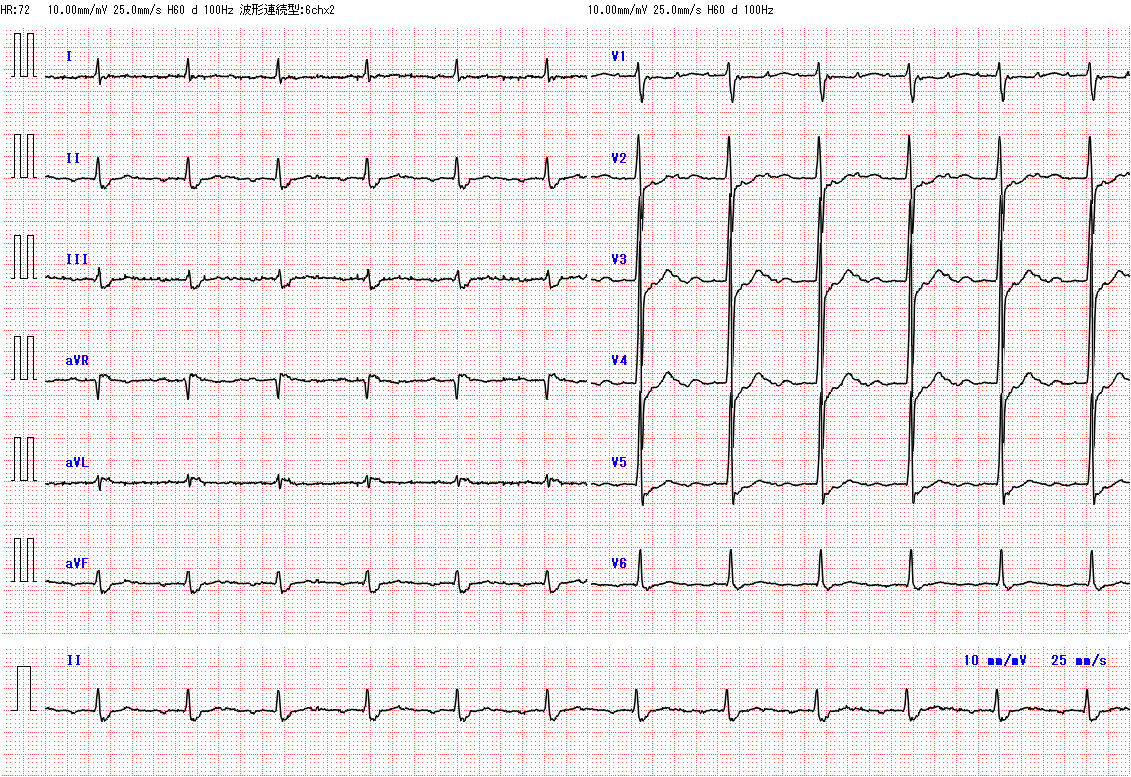
**

**Supplementary Figure 3. ECG on admission**


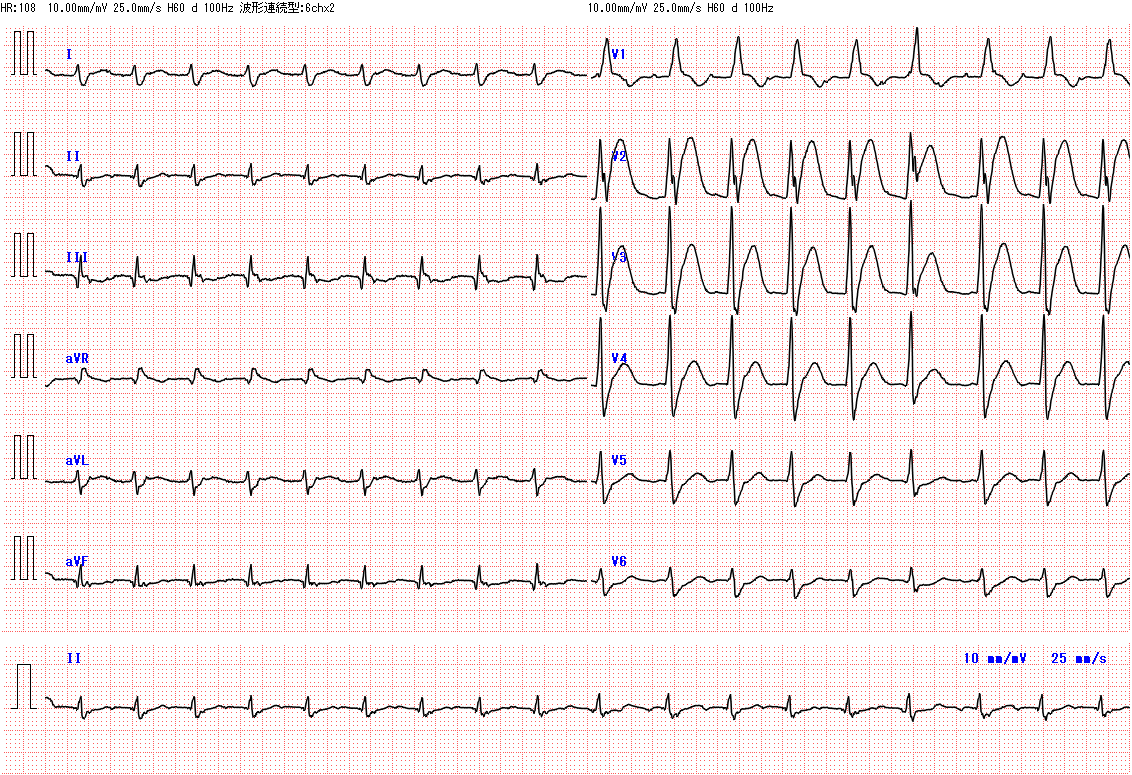


**Supplementary Figure 4. ECG after percutaneous coronary intervention**


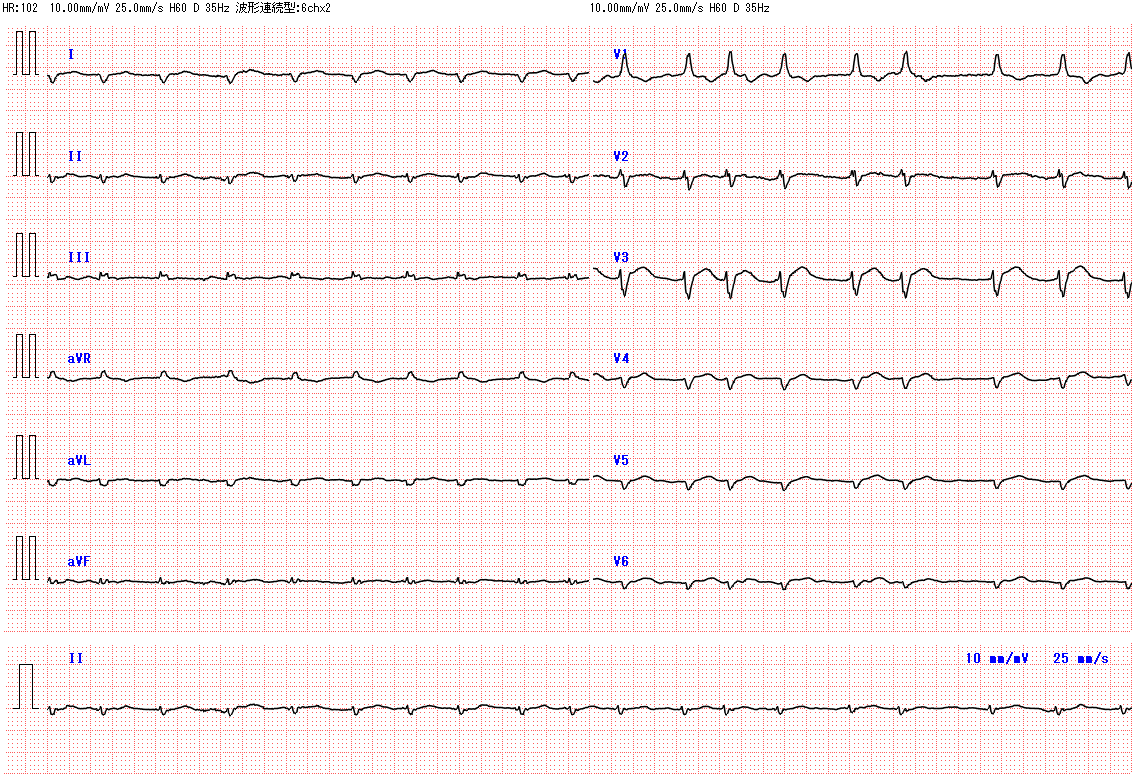


**Supplementary Video Legends**

**Supplementary Video 1.** Coronary angiography of the left coronary artery in the anteroposterior cranial view before PCI, showing acute thrombotic occlusion of the proximal LAD.

**Supplementary Video 2.** Coronary angiography of the left coronary artery in the right anterior oblique view before PCI, showing proximal LAD occlusion.

**Supplementary Video 3.** Right coronary angiography in the LAO view showing chronic total occlusion in the proximal RCA and ipsilateral collateral flow.

**Supplementary Video 4.** Thrombus aspiration of the LAD temporarily restored flow, but re-occlusion occurred shortly afterward.

**Supplementary Video 5.** Balloon pre-dilatation with a 2.0 mm balloon led to temporary restoration of flow, which remained unstable.

**Supplementary Video 6.** Coronary flow after balloon dilatation remained unstable with recurrent re-occlusion.

**Supplementary Video 7.** A 4.0 mm drug-eluting stent was deployed, but incomplete expansion with persistent stent indentation was observed.

**Supplementary Video 8.** Post-stenting balloon dilatation led to hemodynamic collapse, requiring immediate VA-ECMO initiation.

**Supplementary Video 9.** Final AP coronary angiography showing TIMI 3 flow restoration post-PCI, although VA-ECMO support remained necessary.

**Supplementary Video 10.** Final RAO coronary angiography demonstrating restored TIMI 3 flow after PCI, with ongoing need for VA-ECMO support.
